# Diagnostic, prognostic, and immunological roles of FUT8 in lung adenocarcinoma and lung squamous cell carcinoma

**DOI:** 10.1371/journal.pone.0321756

**Published:** 2025-05-15

**Authors:** Zhijun Li, Zhenpeng Zhu, Peng Wang, Chenyang Hou, Lijuan Ren, Dandan Xu, Xiran Wang, Fei Guo, Qingxue Meng, Weizheng Liang, Jun Xue, Xuejun Zhi

**Affiliations:** 1 Hebei North University, Zhangjiakou City, Hebei Province, China; 2 Hebei Key Laboratory of Systems Biology and Gene Regulation, Central Laboratory, The First Affiliated Hospital of Hebei North University, Zhangjiakou City, Hebei Province, China; 3 Department of Bioinformatics, School of Health Care, Changchun Vocational College of Health, Changchun City, Jilin Province, China; 4 Department of Surgery, Hebei Key Laboratory of Systems Biology and Gene Regulation, The First Affiliated Hospital of Hebei North University, Zhangjiakou City, Hebei Province, China; 5 Technology Department, The First Affiliated Hospital of Hebei North University, Zhangjiakou City, Hebei Province, China; 6 Department of Respiratory and Critical Care Medicine, The First Affiliated Hospital of Hebei North University, Zhangjiakou City, Hebei Province, China; Augusta University, TAIWAN

## Abstract

Lung cancer remains the leading cause of malignant tumors worldwide in terms of the incidence and mortality, posing a significant threat to human health. Given that distant metastases typically occur at the time of initial diagnosis, leading to a poor 5-year survival rate among patients, it is crucial to identify markers for diagnosis, prognosis, and therapeutic efficacy monitoring. Abnormal glycosylation is a hallmark of cancer cells, characterized by the disruption of core fucosylation, which is predominantly driven by the enzyme fucosyltransferase 8 (FUT8). Evidence indicates that FUT8 is a pivotal enzyme in cancer onset and progression, influencing cellular glycosylation pathways. Utilizing bioinformatics approaches, we have investigated FUT8 in lung cancer, resulting in a more systematic and comprehensive understanding of its role in the disease’s pathogenesis. In this study, we employed bioinformatics to analyze the differential expression of FUT8 between LUAD and LUSC. We observed upregulation of FUT8 in both LUAD and LUSC, associated with unfavorable prognosis, and higher diagnostic utility in LUAD. GO/KEGG analysis revealed a primary association between LUAD and the spliceosome. Immunologically, FUT8 expression was significantly associated with immune cell infiltration and immune checkpoint activity, with a notable positive correlation with M2 macrophage infiltration. Our analysis of FUT8 indicates that it may serve as a potential biomarker for lung cancer diagnosis and prognosis, and could represent a therapeutic target for LUAD and LUSC immunotherapy.

## Introduction

Global cancer statistics 2022 report that lung malignant tumors account for approximately 12.4% of new cancer cases, ranking first, and the disease is associated with an estimated 1.8 million deaths, representing 18.7% of all cancer-related fatalities [1]. LUAD and LUSC accounting for about 85% of NSCLC case [2]. The 5-year survival rate released for China in 2018 was 19.7% [3]. The high mortality rate of lung cancer is predominantly attributed to the lack of apparent clinical symptoms in the initial stages and the presence of distant metastases at diagnosis, suggesting the need for more effective systemic therapies to enhance long-term survival [2]. The clinical application of targeted therapies and immunotherapies has been on the rise, significantly improving the intermediate survival rates of lung cancer patients, the combination of immunotherapy with chemotherapy significantly improves progression-free survival [4,5]. Moreover, the early administration of immunotherapy can confer substantial advantages [6]. To achieve more targeted treatments, a deeper understanding of tumor characteristics is anticipated to pave the way for robust predictive biomarkers.

Glycosylation, a prevalent modification found across all proteins and lipids [7]. Aberrant glycosylation severs as a hallmark of cancer cells, affecting all stages of tumor progression, including malignant transformation, metastasis and immune evasion. A critical alteration in tumor-associated glycosylation is the modification of core fucosylation, mediated by FUT8 [8,9]. FUT8 expression is widespread in numerous human tissues, with particularly high levels in the brain, placenta, lung, stomach, and small intestine [10]. FUT8 is elevated in a variety of cancers, and affects tumor development and metastasis by regulating key molecules in the tumor microenvironment, such as B7-H3 [11,12]. It was confirmed that FUT8 is a key immunosuppressor and enhance the anti-tumor immune response [11,13–15]. FUT8 is considered to be an important therapeutic target in multiple malignancies that the knockout of FUT8 gene can significantly reduce the migration and proliferation of cancer cell [12,16,17]. FUT8 influences RNA virus replication by regulating IFN-I antiviral response. This finding suggests that FUT8 could be a new target for treating RNA virus-associated diseases, such as hepatitis and some types of cold [18]. The regulatory mechanisms governing FUT8 in the context of cancer are still unclear, despite the identification of several notable correlations. E-calmodulin is considered a substrate for FUT8, and the core fucosylation of E-calmodulin is positively associated with cancer metastasis. Increased FUT8 expression elevates core fucosylation on E-calmodulin, inhibiting its function [19]. Furthermore, core fucoidan glycosylation modulates antibody-dependent cytotoxicity (ADCC) and influences the TGF-β, the epidermal growth factor (EGF) receptor, and α3β1 integrin [8]. The abnormal activity of FUT8 is elevated in tumor cell membrane glycoproteins, which may be related to cancer cell evasion from immune surveillance, the detection of FUT8 levels may serve as a biomarker for evaluating the prognosis and therapeutic response in cancer patients.

## Materials and methods

### FUT8 expression analysis

The Human Protein Atlas (HPA) database (https://www.proteinatlas.org) [20,21] was utilized to investigate the protein expression levels of FUT8 in normal and cancer tissues. The CPTAC dataset from UALCAN cancer database (https://ualcan.path.uab.edu/) [22,23] was employed to investigate the protein expression levels of FUT8 in LUAD and LUSC. Expression levels of FUT8 gene across various cancer tissues were acquired via the TIMER 2.0 (http://timer.cistrome.org/) [24] database’s “Gene_DE” module, enabling the study of differential expression between tumor and adjacent normal tissues. Data were retrieved from TCGA database (https://portal.gdc.cancer.gov), where RNA-seq data for TCGA-LUAD and TCGA-LUSC were organized and normalized to TPM format. Subsequently, RNA-seq data from normal and tumor samples were analyzed to assess differences in FUT8 expression between LUAD/LUSC and para-cancerous tissues. Visualization was conducted using R software and the ggplot2 and stats packages.

### Protein and mRNA expression of FUT8 in LUAD and LUSC in relation to clinicopathologic features

Utilizing the UALCAN web resource (https://ualcan.path.uab.edu/) [22,23], we examined the correlation between RNA transcript and protein levels of FUT8 within the TCGA databases for LUAD and LUSC with various clinicopathological parameters, including cancer stage, age, gender, smoking habits, nodal metastasis status, TP53 mutation status, chromatin modifier status, and pathway status.

### Survival analysis

RNA-Seq data from the TCGA database were downloaded, collated from the TCGA-LUAD and TCGA-LUSC projects via the STAR alignment process, and extracted in TPM format, along with clinical data. Survival analyses for FUT8 in LUAD and LUSC were conducted using the Kaplan-Meier (K-M) method from the survival package, Cox proportional hazards regression to calculate p-values, and to compare overall survival (OS), disease-specific survival (DSS), and progression-free interval (PFI) between high and low FUT8 expression levels. The calculations were performed using both the survminer and ggplot2 packages, and 95% confidence intervals and p-values were visualized. The hazard ratio (HR) was plotted. Statistical significance was defined as *p* < 0.05.

### ROC curves for FUT8 in LUAD and LUSC

ROC curves were employed to assess the diagnostic utility of FUT8 in LUAD and LUSC. The data employed to generate the ROC curves were sourced from the mRNA expression levels of FUT8 in cancerous and matched normal tissues within the TCGA dataset. ROC curves were generated with the R package “pROC” (version 1.17.0.1) [25] and visualized using the “ggplot2” package. Simultaneous calculations were performed for the area under the curve (AUC), critical value, sensitivity, specificity, positive predictive value, negative predictive value, and Youden’s index (YI). This was determined by the AUC’s proximity to 1, with higher values indicating improved diagnostic accuracy. The diagnostic accuracy was classified as low for AUC values between 0.5 and 0.7, moderate for AUC values between 0.7 and 0.9, and high for AUC values of 0.9 or above.

### Protein-protein interaction (PPI) network analyses of FUT8

Ten genes closely associated with FUT8 were identified from STRING (http://cn.string-db.org) [26], and a protein-protein interaction (PPI) network was constructed using a specified threshold. Cystoscape software was utilized for visualization. RNA-seq data from the TCGA database were retrieved and organized for the STAR alignment procedure for TCGA-LUAD and TCGA-LUSC, and were processed into TPM format. Clinical data and log2(value+1) transformations were also prepared. Correlation analysis was performed between the factors identified through STRING and the RNA-seq data, with the results visualized using the ggplot2 package for co-expression heatmap analysis. These findings were then subjected to Spearman’s rank correlation testing.

### Functional enrichment analysis

To investigate the role of FUT8, we retrieved and compiled RNA-seq data derived from the STAR alignment of TCGA-LUAD and TCGA-LUSC projects. These data were normalized into TPM format and categorized into high and low expression groups based on FUT8 expression levels (low expression: 0–50%; high expression: 50–100%). Subsequently, we utilized the DESeq2 package to conduct differential expression analysis on the raw count matrices of the curated public datasets. This resulted in single-gene differential analysis data. We further refined these data using ggplot2 for visualization. The counts matrices of the public datasets were analyzed using standard procedures to identify single-gene differential expression data. This data was filtered for FUT8 and visualized with ggplot2. The filtered genes were subjected to GO, Kyoto Encyclopedia of Genes and Genomes (KEGG), and GSEA [27] for functional annotation. We employed the “clusterProfiler” [28] package to automate the GO/KEGG and GSEA analyses, displaying results via bubble plots and mountain plots using ggplot2.

### Immunoassay of FUT8 in LUAD and LUSC

Utilizing the TCGA database (https://portal.gdc.cancer.gov), correlation data were downloaded using the ssGSEA algorithm available within the GSVA R package (version 1.46.0; Hänzelmann et al., 2013) [29]. Immune infiltration was quantified using markers for 24 immune cells as described in Bindea et al. (2013) [30]. The data resulting from Spearman correlation and variance analysis were visualized using lollipop and scatter plots generated with the ggplot2 package. The enrichment of FUT8 expression in LUAD and LUSC immune-infiltrating cells was assessed using the Wilcoxon rank sum test.

We obtained the harmonized and standardized pan-cancer dataset, TCGA Pan-Cancer (PANCAN; N = 10,535, G = 60,499), from the UCSC database (https://xenabrowser.net/). Subsequently, we extracted expression data from TCGA-LUAD and TCGA-LUSC and excluded all normal samples. Subsequently, we selected samples from primary blood-derived and primary tumor origins, excluded all normal samples, and transformed each expression value using log2(x + 0.001) normalization, based on Pearson correlation analysis and analysis of variance. We analyzed the data using Pearson correlation analysis and analysis of variance to organize expression and correlation data between FUT8 and associated checkpoint genes. Stromal, Immune, and ESTIMATE scores for each patient within each tumor were computed using the R package ESTIMATE based on gene expression data. Infiltration scores for Macrophages_M1 and Macrophages_M2 in LUAD and LUSC were recalculated based on gene expression using the QUANTISEQ method from the R package IOBR. The correlation between these scores and FUT8 expression was investigated using the SangerBox platform. We retrieved data for FUT8 and the four immune checkpoints from the TIMER 2.0 database, organizing it for visualization as dot and radar plots using the ggplot2 package.

### Quantitative real-time PCR

Total RNA was purified by an RNA extraction kit according to the manufacturer’s instructions. The primer sequences were as follows: FUT8, forward 5’-GACAGAACTGGTTCAGCGGAGA-3’ and reverse 5’-GCAGTAGACCACATGATGGAGC-3’; GAPDH, forward 5’-ACCCACTCCTCCACCTTTGA-3’ and reverse 5’-TTGCTGTAGCCAAATTCGTTG-3’.

### Western blotting (WB)

Transfection efficiency was verified by WB assay. The intact protein was separated by SDS‒PAGE and transferred onto a PVDF membrane using a wet transfer method. The membrane was blocked with 5% skim milk powder for 2 h at room temperature and then incubated at 4 °C overnight with primary antibodies specific for FUT8 (mouse monoclonal, Cat No. 66118-1-Ig, 1:1000) or GAPDH (mouse monoclonal, Cat No.60004-1-Ig, 1:5000); GAPDH served as the internal reference. HRP-conjugated goat anti-mouse IgG(H + L) (Cat No.SA00001-1, 1:10000) was used as the secondary antibody. After incubating with the secondary antibody for 2 h at room temperature, the membrane was developed with an enhanced chemiluminescence (ECL) solution.

### Cell culture and transfection

A549 NSCLC cell line was cultured in DMEM medium supplemented with 10% FBS and 1% PS at 37 °C containing 5% CO_2_. Two siRNAs targeting the FUT8 mRNA region and Nc control siRNA were used for FUT8 silencing via transient transfection. The sequences of siRNAs against FUT8 were as follows: 5’-GGACUGCACAAUCGAUACA-3’ (si-1); 5’-CGCAGAUCGACUUGUACGA-3’ (si-2). Cells were seeded in a six-well plate 24 h before transfection. When cells reached 60%-70% confluence, Lipofectamine™ RNAiMAX was utilized to transfect with siRNAs following the manufacturer’s instructions. For validation, 48 h after transfection, total RNA and proteins were extracted for RT-qPCR and WB assays as described above.

### Cell proliferation and invasion assays

Cell proliferation assays were performed with a CCK-8 kit. Cells were harvested 24 h after transfection and then seeded into 96-well plates (3000 cells/well) with six replicates per sample. After 0, 24, 48, 72 and 96 h, 100 µl of 10% CCK-8 serum-free medium was added and cell proliferation was estimated using a microplate reader.

Cell invasion was assessed using a 24-well transwell plate. For the invasion assay, the upper chamber was filled with 200μl serum-free medium and 5 × 10^4^ cells and with Matrigel coated on the upper chamber; the lower chamber was filled with 60μl 20% FBS medium. After 24 h, the cells on the bottom surface were fixed with 4% paraformaldehyde and stained with crystal violet for 20 min.

## Results

### FUT8 expression landscape

**Fig 1:**
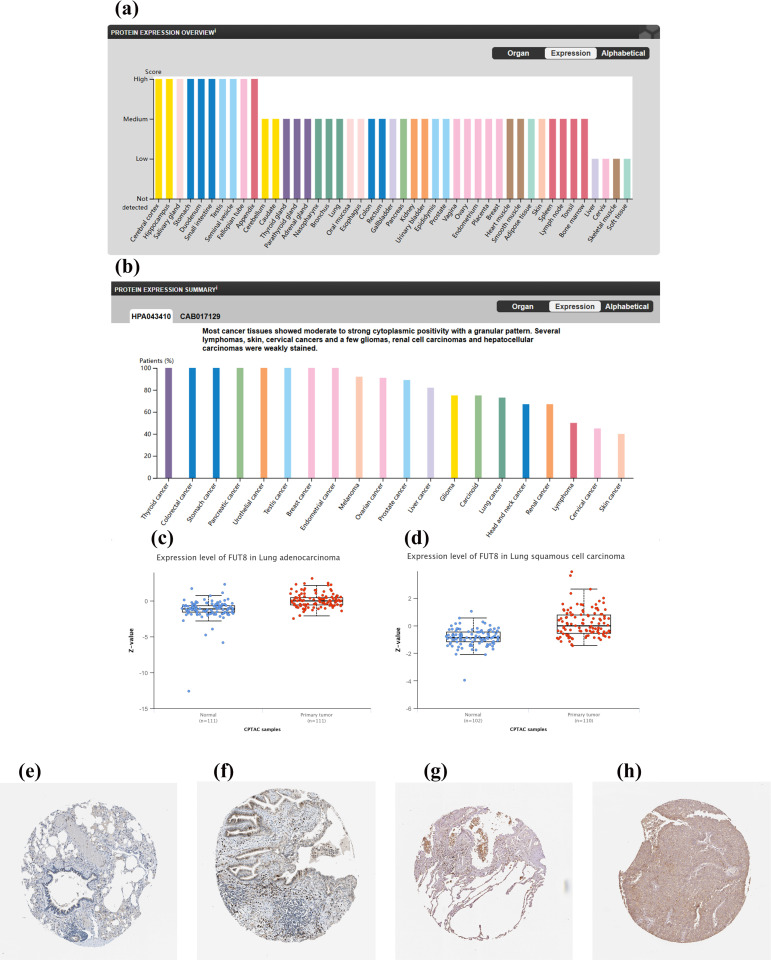
FUT8 protein expression landscape and differences. (a) FUT8 protein expression data in human normal tissues from HPA database. (b) The expression of FUT8 protein in pan-cancer tissues from HPA database. (c) FUT8 protein expression in LUAD. (d) FUT8 protein expression in LUSC. (e, g) Representative IHC images of FUT8 expression in normal lung. (f) Representative IHC images of FUT8 expression in LUAD. (h) Representative IHC images of FUT8 expression in LUSC.

To investigate the expression profile of FUT8, we first searched the HPA database, and the analysis indicated that FUT8 protein expression was particularly high in the cerebral cortex, hippocampus, salivary gland, stomach, duodenum, small intestine, testis, seminal vesicle, fallopian tube, and appendix. Additionally, FUT8 protein expression was also moderate in lung tissue **(Fig 1a**). FUT8 protein expression was evaluated utilizing samples from cancer patients within the HPA database, and was highly expressed across a range of cancers including thyroid cancer, colorectal cancer, stomach cancer, pancreatic cancer, urothelial cancer, testis cancer, breast cancer, endometrial cancer, melanoma, ovarian cancer, prostate cancer, liver cancer, glioma, carcinoid tumors, lung cancer, and others (**Fig 1b**). Sections stained with IHC for FUT8 in normal lung tissue, lung adenocarcinoma, and lung squamous cell carcinoma were retrieved and downloaded from the HPA database (**Fig 1**e–h). FUT8 expression was observed in the cell membrane and within the cytoplasm. Comparatively, the tissue staining from Fig 1e and g, in contrast to LUAD (**Fig 1f**) and LUSC (**Fig 1h**), revealed that the sections of cancer tissues demonstrated more intense staining than the normal tissues (**Fig 1**e,g**).** The analysis of the CPTAC dataset, utilizing UALCAN, revealed that FUT8 expression levels were significantly increased in LUAD and LUSC compared to normal tissue (*p* < 0.001), and the differences were statistically significant (**Fig 1**c,d).

**Fig 2:**
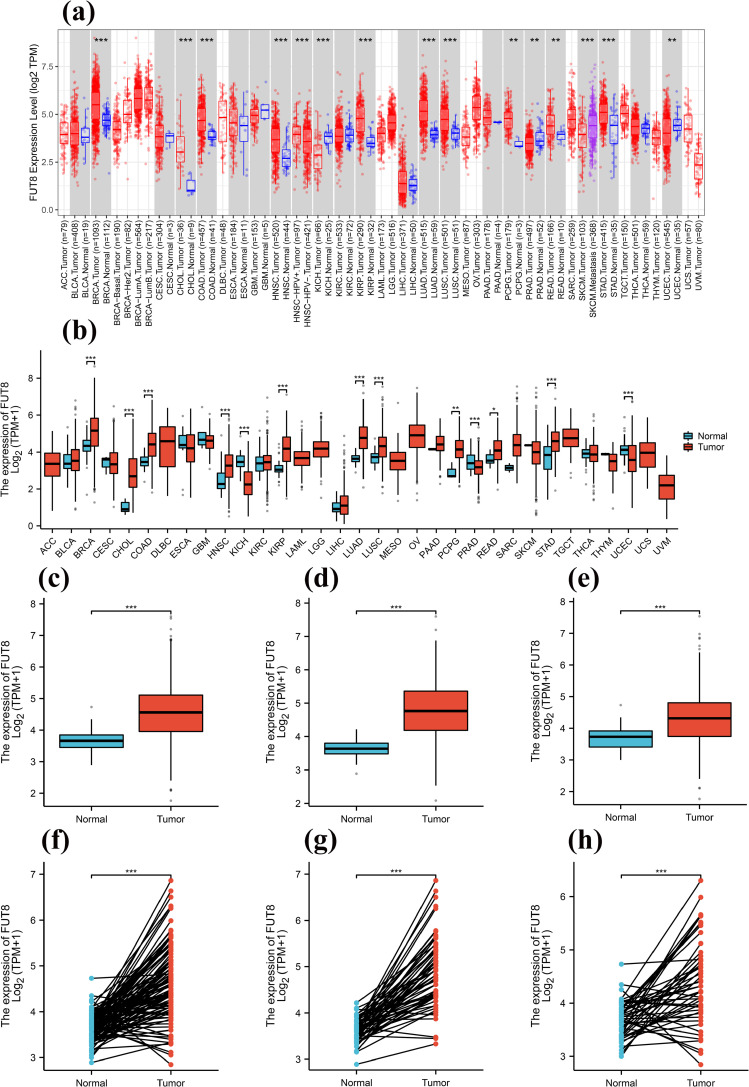
FUT8 mRNA expression landscape and differences. (a) The expression of FUT8 in pan-cancer from TIMER 2.0 database. (b) The expression of FUT8 in pan-cancer from TCGA database. (c, f) FUT8 expression in LUAD LUSC from TCGA database. (d, g) FUT8 expression in LUAD from TCGA database. (e, h) FUT8 expression in LUSC from TCGA database. *p < 0.05, **p < 0.01, ***p < 0.001.

To investigate the expression of FUT8 mRNA, we analyzed date in the Timer 2.0 online database, which showed that FUT8 was widespread expressed in human pan-cancer tissues, with significantly higher expression in BRCA, CHOL, COAD, HNSC, KIRP, LUAD, LUSC and STAD (all *p* < 0.001), PCPG and READ (*p* < 0.01) were expressed higher than normal tissues, significantly lower in KICH (*p* < 0.001), PRAD and UCEC (*p* < 0.01), and higher in metastasis-associated SKCM (*p* < 0.001) than without metastasis, and the differences in expression were all statistically significant (**Fig 2a**). Pairwise analysis of 11,123 tissue pairs from the TCGA database revealed a significant overexpression of FUT8 in BRCA, CHOL, COAD, HNSC, KIRP, STAD, LUAD and LUSC (all *p* < 0.001), PCPG (*p* < 0.01), READ (*p* < 0.05), low expression in KICH, PRAD and UCEC (all *p* < 0.001), and the differences in expression were all statistically significant (**Fig 2b**). Then, we performed expression difference analysis based on 1041 Tumor samples and 108 Normal samples from TCGA-LUADLUSC data, and the results suggested that FUT8 showed high expression in LUAD LUSC (**Fig 2c**), *p*  < 0.001. RNA-seq data from the STAR alignment process of the TCGA-LUAD and TCGA-LUSC projects were downloaded from the TCGA database and normalized to TPM format. Additionally, the corresponding numbered, paired paraneoplastic and cancerous tissue samples from the TCGA-LUADLUSC dataset were extracted for conducting a paired-sample t-test. The results indicated that FUT8 expression in LUADLUSC cancerous tissues was significantly elevated compared to paraneoplastic tissues (**Fig 2f**), *p* < 0.001. The datasets for TCGA-LUAD (539 Tumor cases, 59 Normal cases) and TCGA-LUSC (502 Tumor cases, 49 Normal cases) were retrieved from the TCGA database. The data were normalized using the log2(value+1) transformation. Expression between cancerous and paraneoplastic tissues were analyzed using the aforementioned methodology, along with paired sample t-test. The results of expression difference analysis: FUT8 was significantly higher in LUAD and LUSC than in paracancerous tissues with *p* < 0.001, and the results of paired-sample t-test: FUT8 was significantly higher in cancerous tissues than in paracancerous tissues in both LUAD and LUSC with *p* < 0.001. The appealing results all suggest that FUT8 is differentially expressed in both LUAD (**Fig 2**d,g) and LUSC (**Fig 2**e,h).

This study investigated the correlation between FUT8 expression and clinical parameters in LUAD and LUSC, revealing significantly higher mRNA expression levels compared to the normal group across various tumor stages, age, gender, and smoking status. However, limited data on FUT8 protein expression indicated that FUT8 levels were elevated in various gender groups and exhibited high expression in certain related pathway states, as detailed in S1 and S2 Fig.

### The survival analysis and diagnostic value of FUT8

**Fig 3:**
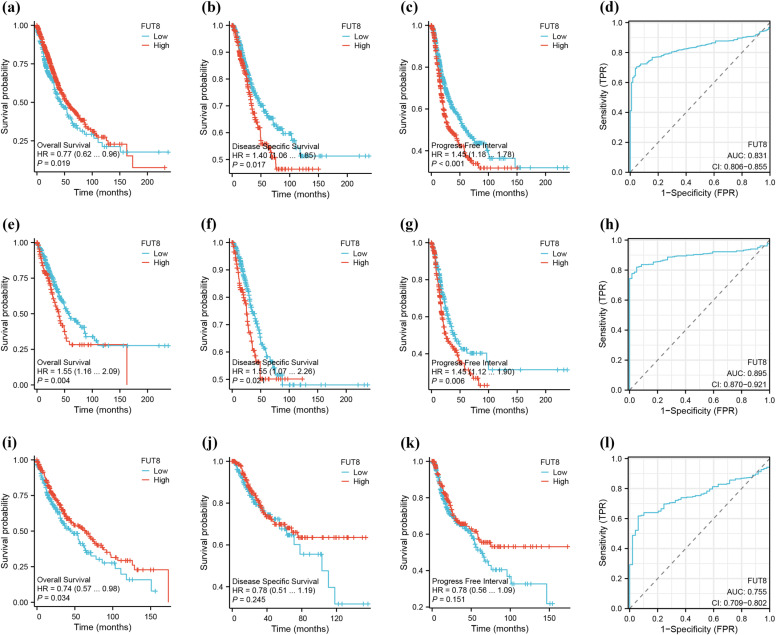
Analysis of prognostic and diagnostic value of FUT8 in LUAD and LUSC in TCGA database. (a-c) Correlation of FUT8 expression with OS, DSS and PFI in LUADLUSC. (d) Clinical ROC analysis of LUADLUSC. (e-g) Correlation of FUT8 expression with OS, DSS and PFI in LUAD. (h) Clinical ROC analysis of LUAD. (i-k) Correlation of FUT8 expression with OS, DSS and PFI in LUSC. (l) Clinical ROC analysis of LUSC. *p < 0.05, **p < 0.01, ***p < 0.001.

We downloaded and collated RNA-Seq data from the TCGA database for the projects TCGA-LUAD, TCGA-LUSC, and TCGA-LUAD/LUSC, using STAR for alignment. The data were extracted in TPM format and associated clinical data, which were log2-transformed (value 1), were used for survival analysis and ROC curve construction for clinical diagnostics. Cox regression proportional hazards testing and survival regression fitting were conducted using the ‘survival’ package, with visualization facilitated by the ‘survminer’ and ‘ggplot2’ packages. Survival analysis was performed on the TCGA-LUAD, TCGA-LUSC, and TCGA-LUAD/LUSC datasets, focusing on OS, DSS, and PFI. Overall Survival analysis showed that the survival prognosis of FUT8 with low expression in LUAD (*p* = 0.004) (**Fig 3e**) was better and statistically significant, whereas high expression in LUAD LUSC (*p* = 0.019) (**Fig 3a**) as well as LUSC (*p* = 0.034) (**Fig 3i**) had a better prognosis, which was statistically significant. Disease Specific Survival analysis showed that high expression of FUT8 in LUAD LUSC (*p* = 0.017) (**Fig 3b**), LUAD (*p* = 0.021) (**Fig 3f**) had a worse prognosis for survival, and the difference was not statistically significant in LUSC (*p* = 0.245) (**Fig 3j**). The results of Progress Free Interval survival analysis showed that FUT8 was highly expressed in LUAD LUSC (*p* < 0.001) (**Fig 3c**) as well as LUAD (*p* = 0.006) (**Fig 3g**) had poor survival prognosis, whereas it was not statistically significant in LUSC (*p* = 0.151) (**Fig 3k**).

Based on clinical data from the TCGA-LUAD, TCGA-LUSC, and TCGA-LUAD LUSC datasets, the data were analyzed using ROC analysis with the pROC package, and the resulting ROC curves were plotted using ggplot2. The results revealed that the AUC values for FUT8 in TCGA-LUAD LUSC was 0.831 (CI 0.806–0.855) (**Fig 3d**), AUC: 0.895 (CI 0.870–0.921) (**Fig 3h**) for TCGA-LUAD, AUC for TCGA-LUSC: 0.755 (CI 0.709–0.802) (**Fig 3l**). All AUC values were greater than 0.7. These results suggest that FUT8 possesses significant clinical diagnostic value and potential in both LUAD and LUSC, compared to LUAD, it demonstrated higher diagnostic value and greater potential for LUAD diagnosis.

### FUT8 interaction analysis

**Fig 4:**
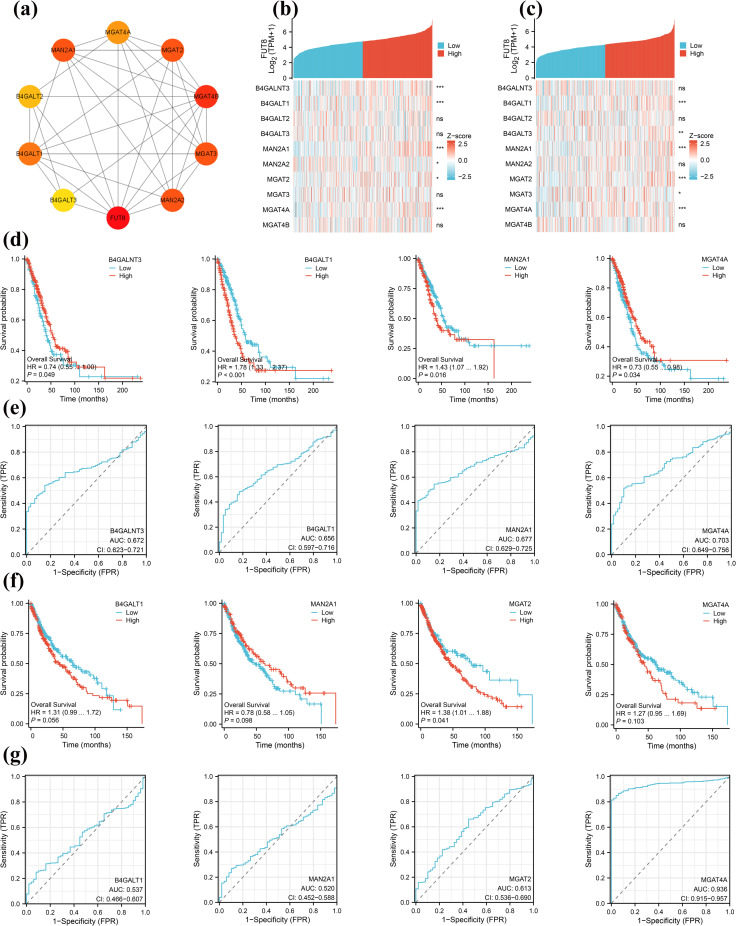
PPI network and related gene expression analysis of FUT8. (a) String interaction network of FUT8. (b) Co expression analysis of string intersection genes in LUAD. (c) Co expression analysis of string intersection genes in LUSC. (d) Correlation of B4GALNT3、B4GALT1、MAN2A1 and MGAT4A expression with OS, in LUAD. (e) Clinical ROC analysis of B4GALNT3、B4GALT1、MAN2A1 and MGAT4A, in LUAD. (f) Correlation of B4GALT1、MAN2A1、MGAT2 and MGAT4A expression with OS, in LUSC. (e) Clinical ROC analysis of B4GALT1、MAN2A1、MGAT2 and MGAT4A, in LUSC.

Ten genes, closely associated with FUT8, were retrieved from the STRING database. A protein-protein interaction (PPI) network was subsequently constructed based on predefined thresholds and visualized using Cytoscape software. The analysis revealed the first nine related genes (**Fig 4a**). These genes were identified as B4GALNT3, B4GALT1, B4GALT2, B4GALT3, MAN2A1, MAN2A2, MGAT2, MGAT3, MGAT4A and MGAT4B. Gene co-expression analysis was conducted between FUT8 and ten genes to investigate their expression in LUAD and LUSC. RNA-Seq data from the TCGA-LUAD, TCGA-LUSC, and TCGA-LUADLUSC projects, processed through the STAR alignment tool, were downloaded from the TCGA database. These data, along with clinical information, were normalized to TPM and subjected to log2 transformation. The Spearman correlation coefficient was then applied to generate a heat map illustrating the co-expression patterns of these genes. In LUAD, B4GALNT3, B4GALT1, MAN2A1 and MGAT4A (all *p* < 0.001) correlated significantly with FUT8 (**Fig 4b**); in LUSC, B4GALT1, MAN2A1, MGAT2 and MGAT4A (all *p* < 0.001) correlated significantly with FUT8 (**Fig 4c**).

Subsequently, overall survival analysis and clinical diagnostic ROC curves were generated for the identified significant correlation factors. The analysis of overall survival data showed that in LUAD, with higher expression levels of B4GALNT3 (*p* = 0.049) and MGAT4A (*p* = 0.034) associated with improved survival prognosis and statistical significance; conversely, lower expression levels of B4GALT1 (*p* < 0.001) and MAN2A1 (*p* = 0.016) were also associated with improved survival prognosis and statistical significance (**Fig 4d**). Within LUSC, with the expression levels of B4GALT1 (*p* = 0.056), MAN2A1 (*p* = 0.098), and MGAT4A (*p* = 0.103) showing no statistically significant differences; however, lower expression of MGAT2(*p* = 0.041) was associated with improved survival prognosis and statistical significance (**Fig 4f**).

ROC analysis results indicated that in LUAD, B4GALNT3 exhibited an AUC of 0.672 (95% CI: 0.623–0.721), B4GALT1, 0.656 (95% CI: 0.597–0.716), MAN2A1, 0.677 (95% CI: 0.629–0.725), and MGAT4A, 0.703 (95% CI: 0.649–0.756) (**Fig 4e**). In LUSC, B4GALT1 demonstrated an AUC of 0.537 (95% CI: 0.466–0.607), MAN2A1, 0.520 (95% CI: 0.452–0.588), MGAT2, 0.613 (95% CI: 0.536–0.690), and MGAT4A, 0.936 (95% CI: 0.915–0.957) (**Fig 4g**). The aforementioned correlation factors possess clinical diagnostic utility in LUAD and LUSC, with MGAT4A demonstrating the highest diagnostic value and potentially serving as a synergistic diagnostic biomarker for FUT8.

### Functional analysis of enrichment of differentially expressed mRNAs for FUT8

**Fig 5:**
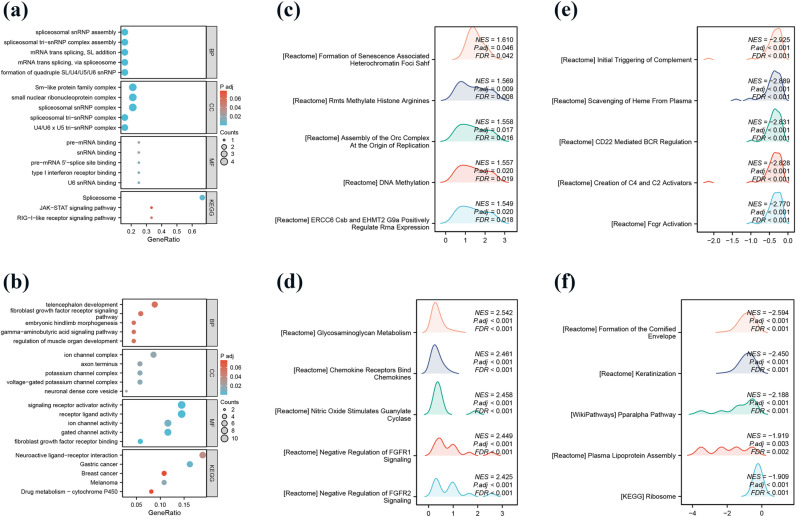
Relevant functional enrichment analysis of FUT8. (a) GO/KEGG analysis of FUT8 in LUAD. (b) GO/KEGG analysis of FUT8 in LUSC. (c, e) top5 pathway with positive and negative correlation in LUAD (d, f) top5 pathway with positive and negative correlation in LUSC.

Using DESeq2 package for TCGA-LUAD, TCGA-LUSC data, the original Counts matrix for differential analysis, FUT8 in LUAD and LUSC into high and low expression of differential analysis. Subsequent data were processed with ggplot2 (version 3.3.6), setting thresholds for log fold change (logFC) to 1 and p-value to 0.05 for filtering, a total of 5353 up-regulated genes and 460 down-regulated genes were screened in LUAD, and a total of 189 up-regulated genes and 434 down-regulated genes were screened in LUSC. The filtered data were then sorted logFC, the top 150 differentially up-regulated genes were selected for GO and KEGG enrichment analyses, and the set of FUT8 differentially up-regulated genes were analyzed by GO/KEGG analysis in LUAD (**Fig 5a**), mainly associated with Spliceosome; GO analysis of differential gene set in LUSC showed (**Fig 5b**), associated with fibroblast growth factor receptor binding, receptor ligand activity, signaling receptor activator activity, and KEGG enrichment analysis (**Fig 5b**) showed that it was mainly related to Neuroactive ligand-receptor interaction.

The sets of FUT8 high and low group differential genes in LUAD and LUSC obtained from the differential analysis of DESeq2 package were analyzed by setting *p* < 0.05, and the resulting statistically significant differential gene sets were taken for GSEA functional enrichment analysis. According to the analysis results, the parameters were set, *p* < 0.05, qvalue<0.25, and the TOP positively and negatively correlated 5 enriched pathways were selected based on the NES values. The GSEA analysis of LUAD showed that NES positive correlation was importantly related to DNA damage and repair, DNA replication, chromatin modification, and gene expression regulation (**Fig 5c**); while NES negative correlation TOP5 was mainly associated with complement activation, signaling, FcγR activation, and associated with disease immunotherapy (**Fig 5e**). GSEA analysis of LUSC showed that NES positive correlation was mainly enriched in the pathways of cell signaling, tissue repair, cell proliferation, differentiation, and migration, etc. (**Fig 5d**), which may be associated with the occurrence and progression of a variety of diseases, including cancers; whereas NES negatively correlates with the main enriched pathways related to lipid metabolism, regulation of inflammatory response, regulation of energy homeostasis and gene expression (**Fig 5f**).

### Correlation of FUT8 expression with immune infiltration

**Fig 6:**
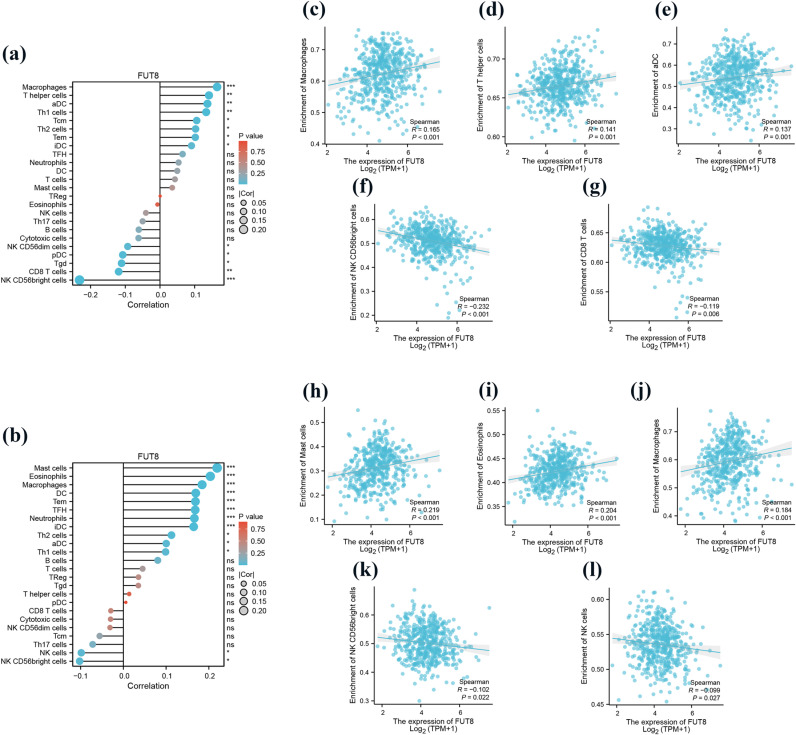
Correlation between FUT8 expression and immune infiltration in LUAD and LUSC. (a) In the bar graph, FUT8 expression was correlated with 24 immune infiltration cells in LUAD. The horizontal axis represents correlations, and the vertical axis represents immune cells. (b) In the bar graph, FUT8 expression was correlated with 24 immune infiltration cells in LUSC. The horizontal axis represents correlations, and the vertical axis represents immune cells. In LUAD, FUT8 expression was positively correlated with (c) Macrophages, (d) T helper cells and (e) aDC cells. In LUAD, FUT8 expression was negatively correlated with (f) NK CD56bright cells and (g) CD8 + T cells. In LUSC, FUT8 expression was positively correlated with (h) Mast cells, (i) Eosinophils, and (j) Macrophages. In LUSC, FUT8 expression was negatively correlated with (k) NK CD56bright cells and (l) NK cells.

The relative abundance of 24 immune cells within LUAD and LUSC was determined using the single sample GSEA (ssGSEA) algorithm, with results demonstrating a correlation between immune cell infiltration levels and FUT8 expression (**Fig 6**a,b). In LUAD, FUT8 expression was mainly positively correlated with Macrophages (cor = 0.165, *p* < 0.001), T helper cells (cor = 0.141, *p* < 0.01), aDC (cor = 0.137, *p* < 0.01) infiltration level (**Fig 6**c–e), and mainly correlated with NK CD56bright cells (cor = -0.232, *p* < 0.001), CD8 T (cor = -0.119, *p* < 0.01) infiltration level was negatively correlated (**Fig 6**f,g). In LUSC, FUT8 expression was mainly positively correlated with the infiltration levels of Mast cells (cor = 0.219, *p* < 0.001), Eosinophils (cor = 0.204, *p* < 0.001), and Macrophages (cor = 0.184, *p* < 0.001) (**Fig 6**h–j), and positively correlated with the infiltration levels of NK CD56bright cells (cor = -0.102, *p* < 0.05), and NK cells (cor = -0.099, *p* < 0.05) infiltration levels were negatively correlated (**Fig 6**k,l).

**Fig 7:**
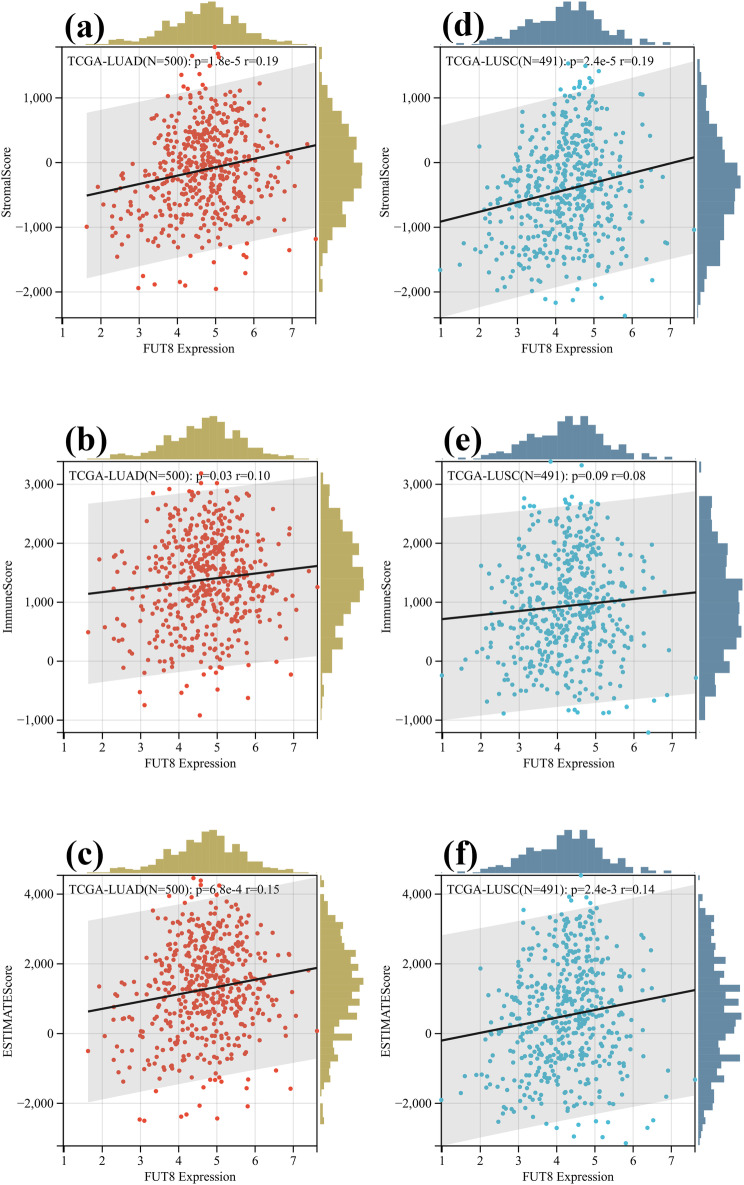
Association between FUT 8 expression and tumor purity. (a-c) FUT8 expression in LUAD and stromal, immune, and estimate scores. (b-f) FUT8 expression in LUSC and stromal, immune, and estimate scores.

We explored the association between FUT8 expression and tumor purity further. The ESTIMATE algorithm was employed to compute stromal, immune, and estimated scores for the relevant tumor samples, derived from the TCGA database, with the correlation between FUT8 expression levels and these scores being evaluated. Our findings revealed a significant and positive correlation between FUT8 expression in LUAD and stromal, immune, and estimate scores (**Fig 7**a–c), whereas in LUSC, FUT8 expression was significantly and positively associated with stromal and estimate scores, but not with immune scores (**Fig 7**d–f). These results suggest a close association between FUT8 expression and tumor purity, as well as the extent of immune cell infiltration within the tumor.

**Fig 8:**
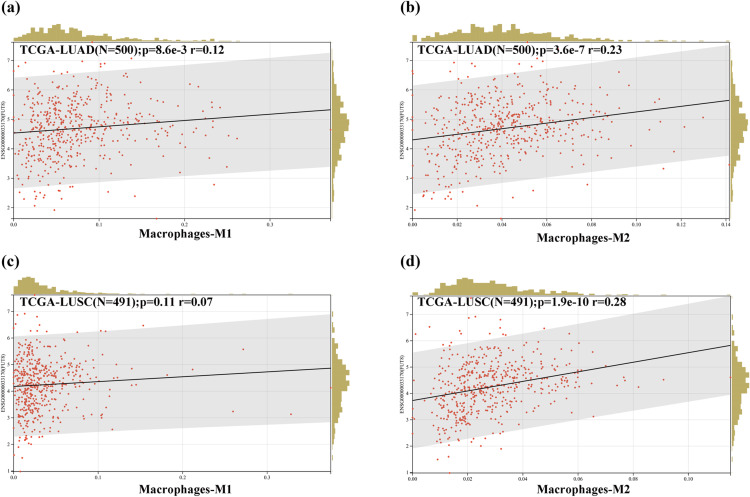
Evaluate infiltration scores for M1 and M2 macrophage subsets in LUAD and LUSC. (a) association between FUT8 expression and M1 macrophage infiltration in LUAD. (b) association between FUT8 expression and M2 macrophage infiltration in LUAD. (c) association between FUT8 expression and M1 macrophage infiltration in LUSC. (d) association between FUT8 expression and M2 macrophage infiltration in LUSC.

For macrophages, the QUANTISEQ method was applied to-evaluate infiltration scores for M1 and M2 macrophage subsets in each patient’s tumor, utilizing gene expression data. Notably, a significant positive association was observed between FUT8 expression and M2 macrophage infiltration in LUAD compared to LUSC (**Fig 8**b,d), and positive between FUT8 expression and M1 macrophage infiltration in LUAD. However, this association was not statistically significant in LUSC (**Fig 8**a,c).

Furthermore, we investigated the composition of immune cell populations in the FUT8-high and FUT8-low expression groups. In LUAD, the FUT8-high expression group exhibited a higher abundance of T helper cells and Th2 cells. Similarly, in LUSC, the FUT8-high expression group displayed increased levels of dendritic cells (DC), mast cells, neutrophils, and macrophages. These findings are illustrated in greater detail in S3 Fig.

### Correlation of FUT8 expression with immune checkpoints and immunotherapy

**Fig 9:**
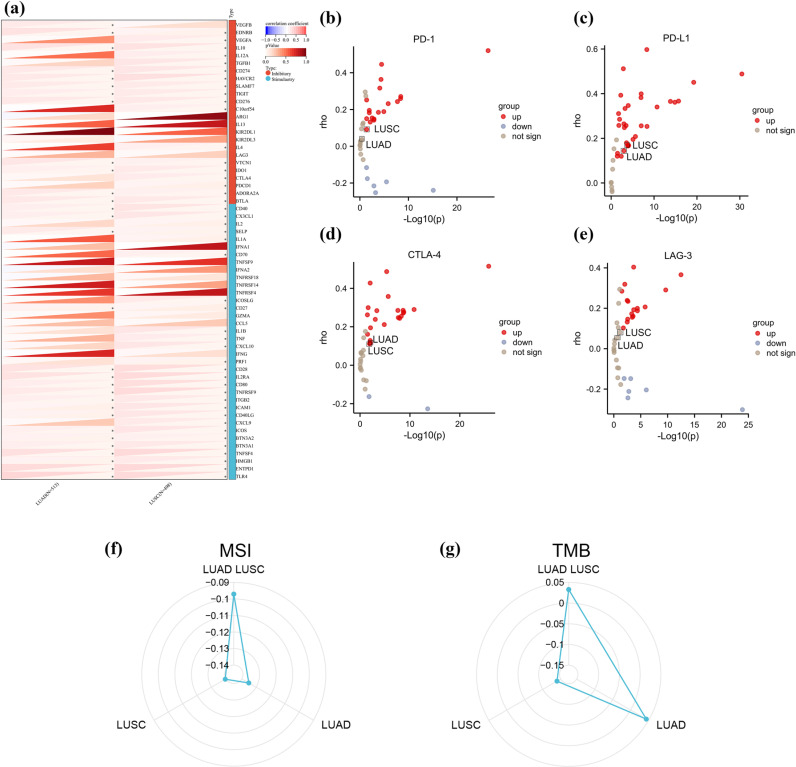
Correlation of FUT8 expression on immune checkpoints and immunotherapy. (a) The correlation of FUT8 with immune checkpoint genes in LUAD and LUSC. The correlation of FUT8 with PD-1(b), PD-L1(c), CTLA-4(d) and LAG-3(e) in the TIMER 2.0 database. (f-g) In LUADLUSC, as well as in LUAD and LUSC, the analysis of the correlation between the expression of FUT8 and microsatellite instability (MSI) and tumor mutational burden (TMB). *p < 0.05, **p < 0.01, ***p < 0.001.

The expression levels of immune checkpoint genes are significantly associated with the therapeutic efficacy of immunotherapies. To investigate the correlations among genes in LUAD and LUSC, we examined the association between FUT8 and immune response-related checkpoint genes as identified through the UCSC database. Our analysis revealed that FUT8 exhibited correlations with the majority of genes examined. Notably, several key genes, including PD-1 (PDCD1), PD-L1 (CD274), and CTLA-4 (CTLA4) displayed positive correlations with FUT8 in LUSC (*p* < 0.05). Conversely, the correlation between FUT8 and LAG-3 was not statistically significant (**Fig 9a**); in LUAD FUT8 was positively associated with PD-L1 *(p* < 0.05), and the correlation with PD-1, CTLA-4, and LAG-3 was not statistically significant (**Fig 9a**). Subsequently, we assessed the association between FUT8 and a panel of immune checkpoint inhibitors such as PD-1, PD-L1, CTLA-4, and LAG-3 using the TIMER 2.0 database. and found that in LUAD, FUT8 was positively correlated with PD-L1 and CTLA-4, while in LUSC, FUT8 was positively correlated with PD-1, PD-L1, CTLA-4, and the correlation with LAG-3 was not statistically significant (**Fig 9**b–e).

Tumor mutational burden (TMB), strongly associated with the response to PD-1/PD-L1 inhibitors, and microsatellite instability (MSI), resulting from DNA mismatch repair defects in tumor tissues, are characterized by the presence of a new microsatellite allele at a tumor locus due to repetitive unit insertions or deletions. This study investigated the relationship between TMB and MSI for FUT8 in LUADLUSC, LUAD, and LUSC, revealing that FUT8 expression levels negatively associated with MSI in LUADLUSC, LUAD, and LUSC, positively with TMB in LUADLUSC and LUAD, and negatively with TMB in LUSC. This correlation was more pronounced in LUAD compared to LUSC (**Fig 9**f,g).

### Knockdown of FUT8 affects the proliferation and invasion of A549 NSCLC cells in vitro

**Fig 10:**
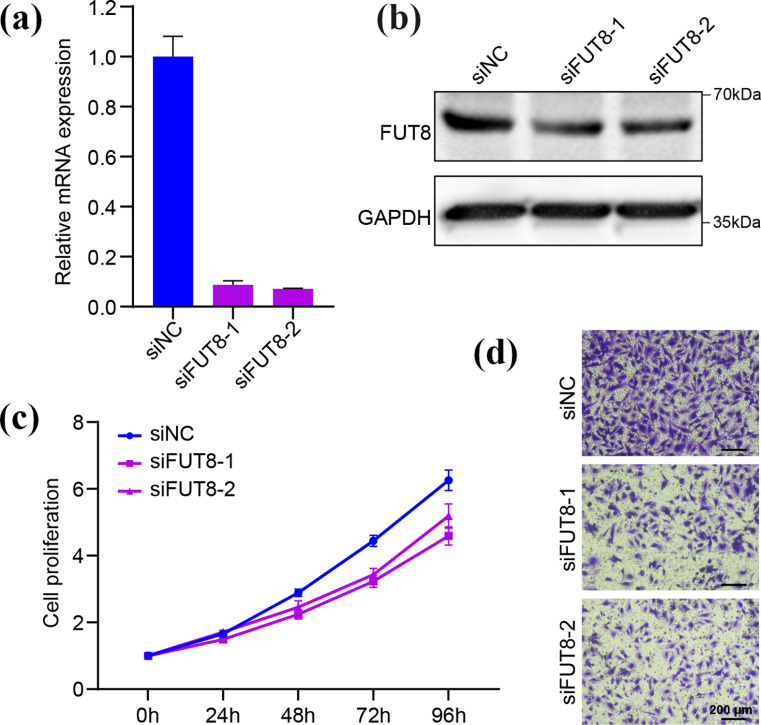
Knockdown of FUT8 affects the proliferation and invasion of A549 NSCLC cells in vitro. (a, b) The transfection efficiency of FUT8 siRNA in A549 cells line evaluated by RT-qPCR and western blot. (c) Proliferation curve of the CCK8 assay between the knockdown and control groups. (d) Transwell assay results between the knockdown and control groups.

We further explored the function of FUT8 in NSCLC cells, we transfected siRNA into NSCLC cells to knock down FUT8 expression. The efficiency of transfection was then verified at the mRNA and protein levels using RT‒qPCR and WB assays, respectively (**Fig 10**a,b). The CCK-8 assay showed that NSCLC cells transfected with si-FUT8 exhibited significantly decreased proliferation compared with those in the control group (si-NC) (**Fig 10c**). In the transwell assay, the knockdown of FUT8 significantly inhibited the invasion of A549 NSCLC cells (**Fig 10d**).

## Discussion

Advancements in molecularly targeted therapies, immune checkpoint inhibitors (ICIs), and anti-angiogenic agents have significantly enhanced systemic treatment options for lung malignant tumors, leading to improved patient prognosis [31]. Nonetheless, considering the occurrence of immune-related adverse events, it is crucial to tailor treatment plans to individual patient conditions and monitor their progress vigilantly. Consequently, there is an imperative need to identify additional immune-related genes to enhance prognosis further.

The fucosyltransferase family constitutes a class of enzymes that catalyze the addition of fucose to glycoproteins and glycolipids. Among FUTs, FUT8 is of particular interest due to its direct association with tumors [32–34]. Elevated fucosylation levels have been implicated in malignant cell transformation, associated with a range of abnormal events during cancer progression, including uncontrolled cell proliferation, tumor cell invasion, angiogenesis, metastasis, immune evasion, and therapeutic resistance [8,35].

We employed bioinformatics to investigate the correlation between FUT8 expression, clinical significance, biological function, and immune infiltration in LUAD and LUSC. Subsequently, our database analysis revealed that FUT8 in LUAD and LUSC were significantly elevated compared to normal tissue, and FUT8 predominantly resides in the cell membrane and cytoplasm. A correlation exists between FUT8 expression and clinical features. Elevated FUT8 expression modulates multiple signaling pathways, potentially impacting cell transformation, proliferation, and contributing to tumor progression. Our findings indicated that FUT8 expression was significantly associated with patient prognosis, with particularly high diagnostic efficacy in LUAD cases. We find that MGAT4A exhibited a superior diagnostic value. Functional enrichment analysis of FUT8-related genes suggested that in LUAD, FUT8 is primarily associated with the spliceosome, a pivotal component in eukaryotic gene expression. GO analysis of the LUSC differential gene set indicated associations with fibroblast growth factor receptor binding, ligand-receptor activity, and receptor activator signaling. KEGG enrichment analysis further suggested a primary involvement in neuroactive ligand-receptor interactions. GSEA analysis in LUAD revealed a significant correlation of NES with pathways involving DNA damage and repair, DNA replication, chromatin modification, gene expression regulation and signaling. GSEA analysis in LUSC demonstrated that NES associated with pathways such as cell signaling, potentially implicated in the development and progression of numerous diseases, including cancer, lipid metabolism, inflammatory response regulation, energy homeostasis, and gene expression. Finally, we investigated the correlation between FUT8 and immune response in LUAD and LUSC. We observed that FUT8 was associated with most immune cell types, including macrophages, NK cells, neutrophils, and T cells. FUT8 expression was significantly associated with tumor purity and the extent of infiltrative immune cells. Elevated FUT8 expression was detected in macrophages and T cells, indicating a higher degree of enrichment. Additionally, FUT8 expression exhibited a significant positive correlation with M2 macrophage infiltration. FUT8 was also associated with several immune checkpoint genes. In LUSC, including CTLA-4, PD-1 and PD-L1. In LUAD, FUT8 expression was associated with PD-L1.

Based on the results of the immune correlation analysis, a robust association was observed between FUT8 and immune cell populations. Correlations between immune cells and lung cancer types exhibit variability, with macrophages predominantly associated with LUAD and LUSC. Macrophages exhibit robust phagocytic capacity, and are pivotal in the pathogenesis of cancer, atherosclerosis, and inflammatory diseases [36]. Macrophages are stratified into two subtypes primarily on the basis of activation status, functional profiles, and cytokine secretion. The M2 subtype of macrophages is characterized by its induction of immunosuppression, engagement in pro-carcinogenic activities, and facilitation of tumor growth and metastasis [37–39]. TAMs, within TME, typically exert suppressive effects on tumor cells through the secretion of cytokines that elicit direct cytotoxicity and antibody-dependent immune responses [40]. TAMs exhibit dynamic behavior, enabling interconversion in response to alterations within the tumor microenvironment or therapeutic manipulations [41]. Prolonged exposure to the TME can induce the transformation of M1 macrophages into M2 macrophages. These TAMs are subsequently recruited by tumor cells to express factors which facilitate tumor growth and metastasis, promote angiogenesis, and suppress the immune response [40].

We identified a significant negative correlation between LUAD, LUSC, and CD56bright NK cells. NK cells, innate immune lymphocytes, are pivotal in defense against infectious agents and cancer. Conventionally, CD56dim NK cells are considered key mediators of antitumor immunity, while CD56bright cells participate in immune regulation. NK cells’ cytotoxic response against tumor cells encompasses a series of events, including cell recognition, coupler formation, immune synapse establishment, cytotoxic particle assembly, and targeted delivery [42]. NK cells exhibit diverse cell surface receptors, which can either activate or inhibit effector functions, such as cytotoxicity and cytokine secretion. Furthermore, NK cell-mediated cytotoxicity and cytokine secretion can modulate the functions of other innate immune cells, including dendritic cells, macrophages, and neutrophils [43]. Research indicates that decreased NK cell-mediated cytotoxicity correlates with an increased risk of various cancers, whereas enhanced NK cell cytotoxicity is linked to a reduced cancer risk [44,45].

Moreover, mast cells and eosinophils demonstrated a strong positive correlation with FUT8 expression in LUSC. Eosinophils, a white blood cell crucial for the immune system, are involved in numerous inflammatory and allergic processes. Elevated levels of eosinophils are often linked to a poorer prognosis in certain cancers [46,47]. Abnormal proliferation and activation of mast cells in specific cancers may significantly contribute to cancer development, progression, and metastasis. Studies indicate that mast cells can secrete diverse inflammatory mediators conducive to tumor growth and angiogenesis [48,49]. The interplay between immune cells and cancer is complex and multifaceted.

Immune checkpoint inhibitors (ICIs) have significantly advanced cancer treatment as a precision therapeutic modality. By targeting specific checkpoint molecules within the immune system, these agents alleviate the suppressive effect of tumor cells on immune cells, thereby enhancing immune-mediated tumor cell destruction [50]. In our investigation of FUT8, we identified correlations between FUT8 expression and numerous immune checkpoints in LUSC, including the predominant inhibitory checkpoints PD-1, PD-L1, and CTLA-4. Clinically, ICIs such as anti-CTLA-4, anti-PD-1, PD-L1, and the novel immune checkpoint inhibitor LAG-3 have demonstrated efficacy across various cancer types, resulting in improved patient outcomes [51,52]. These agents act by augmenting the immune system, triggering T-cell activation, and inducing tumor cell apoptosis. Notably, ICIs can activate diverse cell types within the immune system. These distinct cell types collaborate to combat tumors. T cells commonly express receptors and ligands such as CTLA-4, PD-1, and PD-L1 [53]. PD-1 and its ligand PD-L1 may facilitate tumor progression by evading immune surveillance. Tumors with increased PD-L1 expression protect themselves from destruction by cytotoxic CD8 + T cells [54,55]. Consequently, inhibition of either PD-1 or PD-L1 enhances anti-tumor immune responses.

FUT8 plays an important role in tumor immunology, mainly by regulating the glycosylation state of the surface glycoprotein of tumor cells to affect the immune escape and immunotherapy effect of tumors. FUT8-mediated core fucosylation modification of the B7H3 protein promotes immune escape in breast cancer and triple-negative breast cancer (TNBC) [14]. FUT8 expression is closely related to changes in the TME. For example, FUT8 regulates the cancer-promoting capacity of cancer-associated fibroblasts CAFs in NSCLC [56]. Inhibition of core fucose-transferase Fut8 by gene ablation or drug inhibition reduces cell surface expression of PD-1 and enhances T cell activation, resulting in more effective tumor eradication [57]. In 2023, the Soochow University team developed a small molecule inhibitor of FUT8, FDW028, promoted lysosomal degradation of B7-H3 through fucosylation and CMA pathway, and showed strong anti-tumor activity [11]. FUT8 expression and activity are up-regulated in various human cancers. FUT8 mediates the glycosylation of PD-L2, and glycosylation modification stabilizes PD-L2 by blocking ubiquitin-dependent lysosomal degradation, thereby promoting its binding to PD-1 and immune escape [13]. In animal models, inhibition of Fut8 activity by gene knockout, RNA interference, and small analogue inhibitors resulted in reduced tumor growth/metastasis, downregulation of the immune checkpoint molecules PD-1, PD-L1/2, and B7-H3, and reversal of the inhibited state of the tumor microenvironment [58]. FUT8 plays a key role in the progression of head and neck squamous cell carcinoma (HNSCC) by mediating abnormal N-glycosylation of SEMA7A, which this study suggests causes CD8 + T cells to differentiate into a depleted state, forming an immunosuppressive microenvironment that is resistant to immunotherapy. In addition, glycosylated SEMA7A is involved in regulating cell-extracellular matrix interactions and promoting tumor growth and metastasis [15].

## Conclusion

By examining the expression patterns of FUT8, this study revealed elevated levels of FUT8 in LUAD and LUSC. This upregulation is associated with unfavorable survival outcomes, contributes to diagnostic accuracy, and is implicated in the progression of cancer. Regarding tumor immunity, this study demonstrated a correlation between high FUT8 expression and tumor infiltration. Notably, macrophages, particularly M2-polarized macrophages, exhibited a positive association with FUT8 levels. It was proved by experiments that knocking down FUT8 could inhibit the proliferation and invasion of tumor cells，suggesting that FUT8 could serve as a diagnostic and prognostic biomarker for LUAD and LUSC and as a therapeutic target for immunotherapy.

## Supporting information

S1 Fig mRNA expression of FUT8 in LUAD and LUSC in relation to clinicopathologic features. (a) expression of FUT8 in LUAD based on individual cancer stages; (b) expression of FUT8 in LUSC based on individual cancer stages; (c) expression of FUT8 in LUAD based on patient’s age; (d) expression of FUT8 in LUSC based on patient’s age; (e) expression of FUT8 in LUAD based on patient’s gender; (f) expression of FUT8 in LUSC based on patient’s gender; (g) expression of FUT8 in LUAD based on patient’s smoking habits; (h) expression of FUT8 in LUSC based on patient’s smoking habits; (i) expression of FUT8 in LUAD based on nodal metastasis status; (j) expression of FUT8 in LUSC based on nodal metastasis status; (k) expression of FUT8 in LUAD based on TP53 muation status; (l) expression of FUT8 in LUSC based on TP53 muation status.
(TIF)

S2 Fig Protein expression of FUT8 in LUAD and LUSC in relation to clinicopathologic features. (a) expression level of FUT8 in LUAD based on patient’s gender; (b) expression level of FUT8 in LUSC based on patient’s gender; (c) expression level of FUT8 in LUAD based on Chromatin Modifier status; (d) expression level of FUT8 in LUSC based on Chromatin Modifier status; (e) expression of FUT8 in LUAD based on p53/Rb-related pathway status; (f) expression level of FUT8 in LUSC based on p53/Rb-related pathway status; (g) expression level of FUT8 in LUAD based on SWI-SNF complex status; (h) expression level of FUT8 in LUSC based on SWI-SNF complex status; (i) expression level of FUT8 in LUAD based on mTOR pathway status; (j) expression level of FUT8 in LUSC based on mTOR pathway status; (k) expression level of FUT8 in LUAD based on RTK pathway status; (l) expression level of FUT8 in LUSC based on RTK pathway status.
(TIF)

S3 Fig Composition of immune cell populations in the FUT8-high and FUT8-low expression groups in LUAD and LUSC. (a) enrichment score of NK CD56bright cell in LUAD; (b) enrichment score of pDC in LUAD; (c) enrichment score of CD8 T cell in LUAD; (d) enrichment score of NK CD56dim cell in LUAD; (e) enrichment score of B cell in LUAD; (f) enrichment score of T helper cell in LUAD; (g) enrichment score of Th2 cell in LUAD; (h) enrichment score of Eosinophils in LUSC; (i) enrichment score of Mast cell in LUSC; (j) enrichment score of DC in LUSC; (k) enrichment score of Macrophages in LUSC; (l) enrichment score of Neutrophils in LUSC; (m) enrichment score of TFH in LUAD; (n) enrichment score of Tem in LUSC; (o) enrichment score of iDC in LUSC; (p) enrichment score of aDC in LUSC; (q) enrichment score of NK cell in LUSC.
(TIF)
