# Supplementary figures and images for: Diagnostic, prognostic, and immunological roles of FUT8 in lung adenocarcinoma and lung squamous cell carcinoma
Source: PLoS One. 2025 May 15;20(5):e0321756. doi: 10.1371/journal.pone.0321756 (PMC12080848; doi:10.1371/journal.pone.0321756)

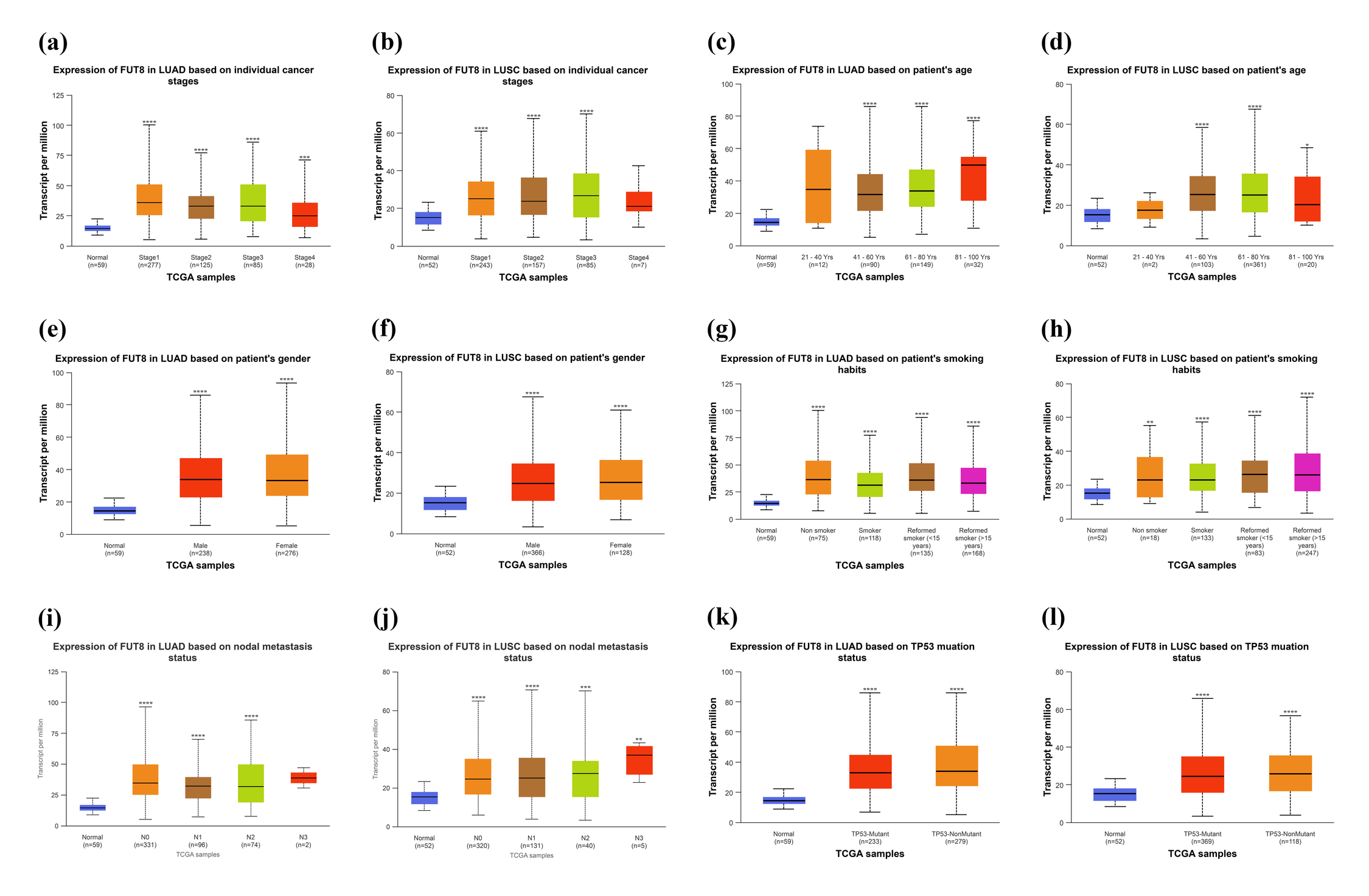

Supplement: S1 Fig — (a) expression of FUT8 in LUAD based on individual cancer stages; (b) expression of FUT8 in LUSC based on individual cancer stages; (c) expression of FUT8 in LUAD based on patient’s age; (d) expression of FUT8 in LUSC based on patient’s age; (e) expression of FUT8 in LUAD based on patient’s gender; (f) expression of FUT8 in LUSC based on patient’s gender; (g) expression of FUT8 in LUAD based on patient’s smoking habits; (h) expression of FUT8 in LUSC based on patient’s smoking habits; (i) expression of FUT8 in LUAD based on nodal metastasis status; (j) expression of FUT8 in LUSC based on nodal metastasis status; (k) expression of FUT8 in LUAD based on TP53 muation status; (l) expression of FUT8 in LUSC based on TP53 muation status. (TIF) [file pone.0321756.s001.tif]

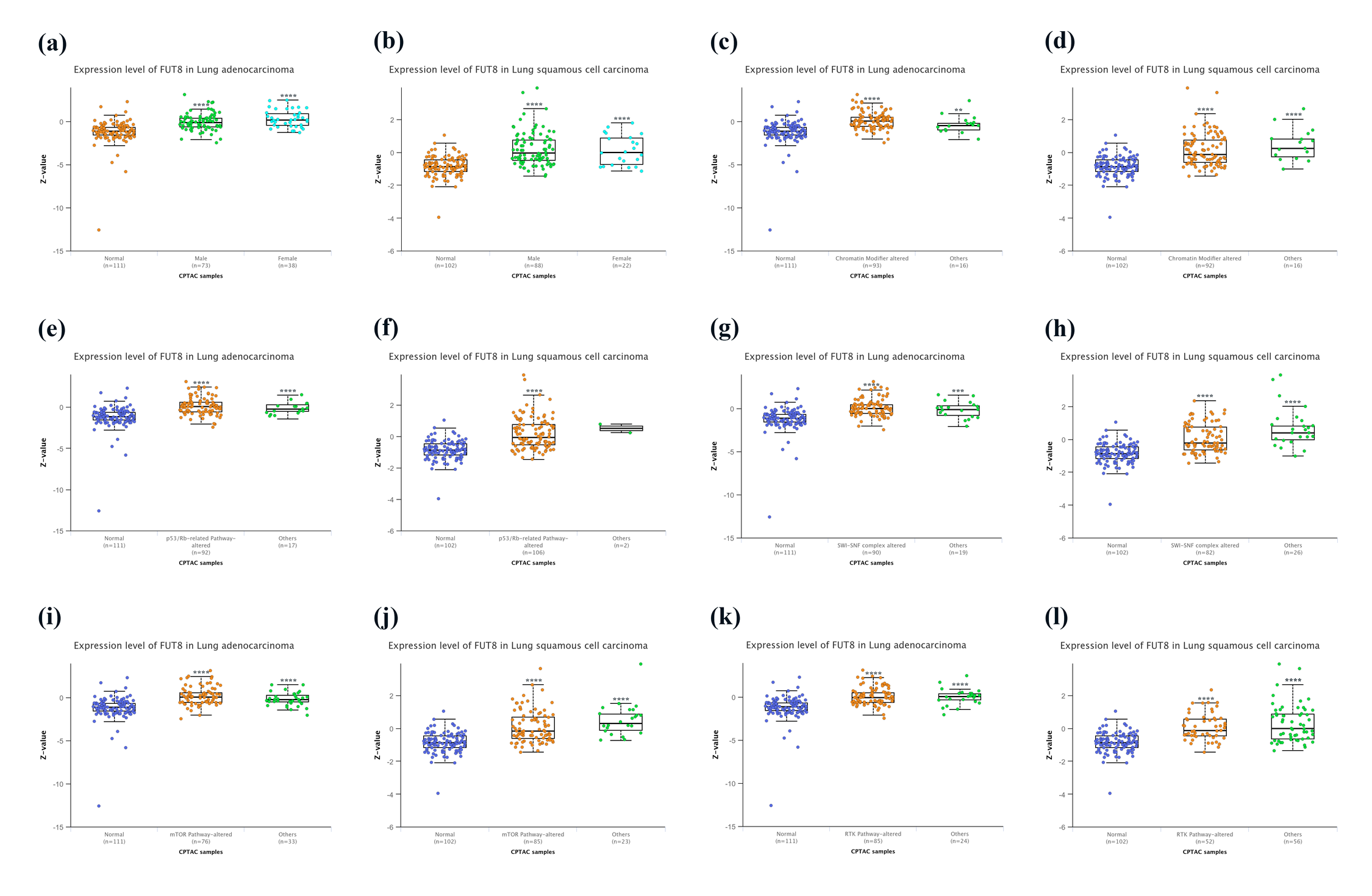

Supplement: S2 Fig — (a) expression level of FUT8 in LUAD based on patient’s gender; (b) expression level of FUT8 in LUSC based on patient’s gender; (c) expression level of FUT8 in LUAD based on Chromatin Modifier status; (d) expression level of FUT8 in LUSC based on Chromatin Modifier status; (e) expression of FUT8 in LUAD based on p53/Rb-related pathway status; (f) expression level of FUT8 in LUSC based on p53/Rb-related pathway status; (g) expression level of FUT8 in LUAD based on SWI-SNF complex status; (h) expression level of FUT8 in LUSC based on SWI-SNF complex status; (i) expression level of FUT8 in LUAD based on mTOR pathway status; (j) expression level of FUT8 in LUSC based on mTOR pathway status; (k) expression level of FUT8 in LUAD based on RTK pathway status; (l) expression level of FUT8 in LUSC based on RTK pathway status. (TIF) [file pone.0321756.s002.tif]

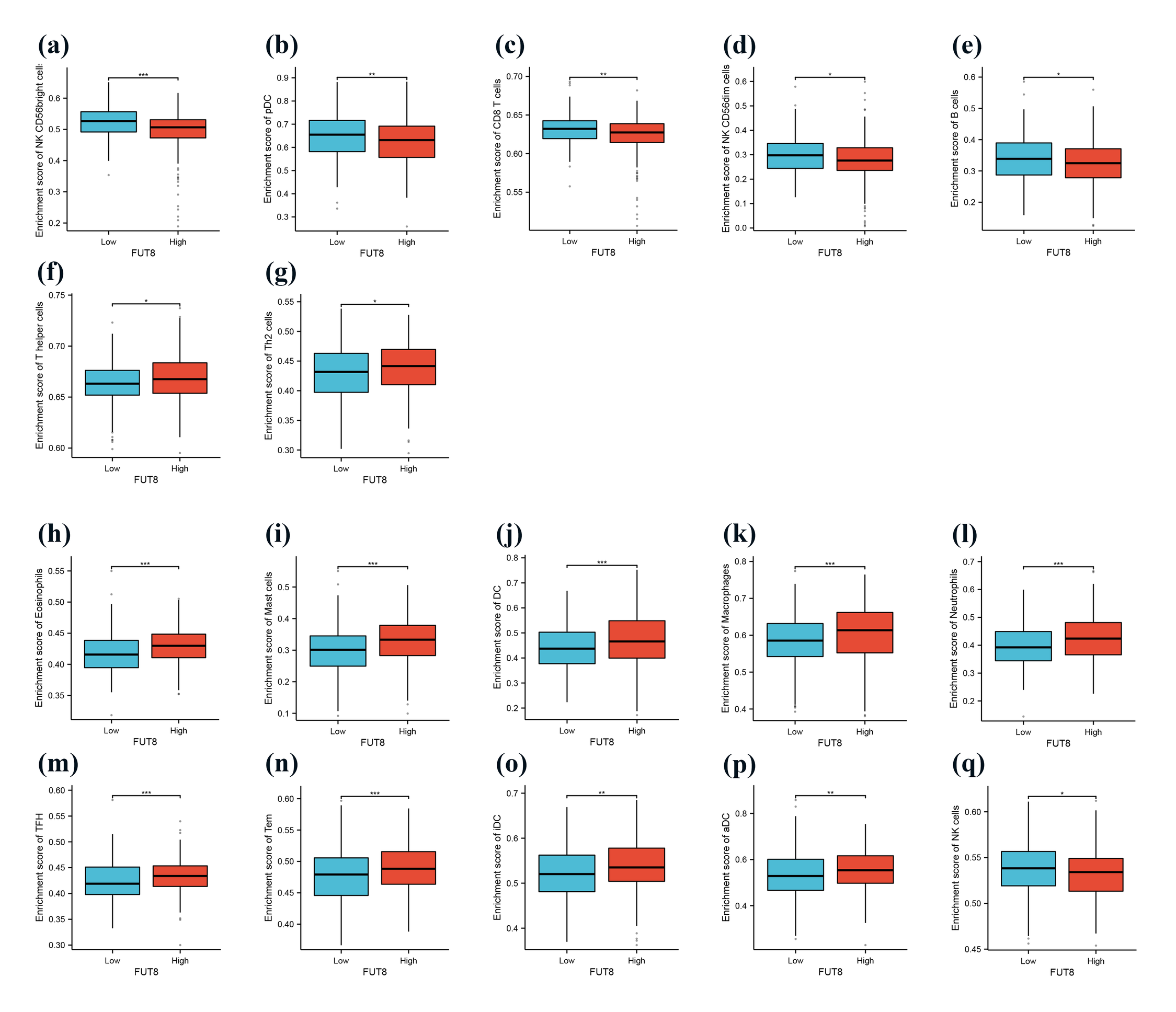

Supplement: S3 Fig — (a) enrichment score of NK CD56bright cell in LUAD; (b) enrichment score of pDC in LUAD; (c) enrichment score of CD8 T cell in LUAD; (d) enrichment score of NK CD56dim cell in LUAD; (e) enrichment score of B cell in LUAD; (f) enrichment score of T helper cell in LUAD; (g) enrichment score of Th2 cell in LUAD; (h) enrichment score of Eosinophils in LUSC; (i) enrichment score of Mast cell in LUSC; (j) enrichment score of DC in LUSC; (k) enrichment score of Macrophages in LUSC; (l) enrichment score of Neutrophils in LUSC; (m) enrichment score of TFH in LUAD; (n) enrichment score of Tem in LUSC; (o) enrichment score of iDC in LUSC; (p) enrichment score of aDC in LUSC; (q) enrichment score of NK cell in LUSC. (TIF) [file pone.0321756.s003.tif]
